# Early adherence to biofeedback training predicts long-term improvement in stroke patients: A machine learning approach

**DOI:** 10.1371/journal.pone.0336503

**Published:** 2025-11-13

**Authors:** Nandini Sengupta, Rezaul Begg, Aravinda S. Rao, Soheil Bajelan, Catherine M. Said, Lisa James, Marimuthu Palaniswami

**Affiliations:** 1 Department of Electrical and Electronic Engineering, The University of Melbourne, Parkville, Victoria, Australia; 2 Institute for Health and Sport, Victoria University, Melbourne, Victoria, Australia; 3 Melbourne School of Health Sciences, The University of Melbourne, Parkville, Victoria, Australia; 4 Physiotherapy Department, Western Health, St. Albans, Victoria, Australia; 5 Australian Institute for Musculoskeletal Science (AIMSS), St. Albans, Victoria, Australia; 6 Physiotherapy Department, Austin Health, Heidelberg, Victoria, Australia; University of Bucharest, Faculty of Biology, ROMANIA

## Abstract

Biofeedback-based treadmill training generally involves 10 or more sessions to assess its effectiveness during stroke rehabilitation. Improvements are seen in some patients during the assessment, while others do not progress. Our aim in this study is to determine (i) if signs of progress are evident from the initial training session and (ii) whether quantitative measurements between consecutive training sessions can guide interventions for non-progressing patients. The study analyzes Minimum Foot Clearance (MFC) data from 15 stroke patients during their baseline and second training sessions to predict outcomes in the post-assessment phase. Based on early biofeedback training data, we propose a novel approach using cosine similarity (CS), correlation coefficient (CC) and cross-correlation distance (XCRD) measures to predict post-assessment improvements in stroke patients. We also introduce a new real-time adherence assessment metric (RAAM) metric to quantify improvements in adherence to feedback between consecutive training sessions, enabling more targeted interventions. The proposed approach using CS, CC and XCRD adherence indicators demonstrates 100% accuracy in predicting improvement during post-assessments. The results show that patients with MFC values dissimilar to their baseline while adhering to targeted feedback are more likely to improve. The work also indicates that patients who don’t show significant overall improvement may benefit from extended training periods, suggesting the potential for personalized rehabilitation strategies.

## 1 Introduction

Millions of people worldwide experience the life-altering impact of strokes every year [1]. Among the primary concerns for stroke survivors is the heightened risk of tripping falls [2]. Compared to age- and gender-matched controls, stroke patients are 150% more likely to experience falls [3,4]. Research indicates that nearly half of stroke survivors living at home will encounter a fall within 12 months, with a significant portion experiencing multiple falls [5]. Mitigating this concern requires gait training interventions; however, assessing their effectiveness poses several challenges.

Multiple training sessions and clinical evaluations are necessary, which incur time, labour, and cost. A standardized intervention may not consistently deliver ideal outcomes for every stroke patient, necessitating adjustments to the initial approach to ensure progress towards improvement. Therefore, it becomes essential to identify crucial patterns during the training sessions that can discern the progression of training, particularly for patients who are not likely to experience improvement. Predicting the likelihood of improvement or no improvement following initial training sessions would be beneficial, enabling more tailored interventions.

A higher likelihood of falls is seen among stroke survivors with impaired gait dynamics [2]. Minimum Foot Clearance (MFC) represents the smallest vertical gap between the foot and the walking surface during the mid-swing phase of gait. MFC at mid-swing is a vital factor in predicting tripping incidents [6–8] with low MFC leading to unexpected and destabilizing foot-ground contacts [9]. Stroke survivors who struggle to step over relatively low surface irregularities, approximately 4 cm in height, face a increased fall risk [10], and often demonstrate lower and highly variable MFC control across multiple gait cycles [11]. Additionally, the foot’s horizontal velocity peaks at MFC, resulting in a powerful impact when contacting an obstacle [12]. When an obstacle is hit with such a high momentum; the likelihood of a fall increases significantly [13]. The body’s capacity to recover from sudden disruptions is compromised by the force of the impact [14], which has significant clinical implications. Therefore, increasing MFC height and maintaining consistent ground clearance to reduce variability in MFC height are essential to minimize fall risk [15].

**Fig 1:**
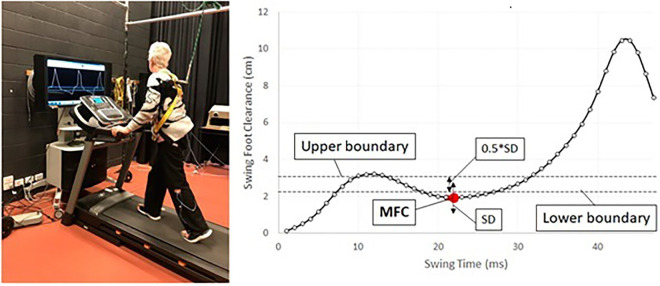
The diagram represents a person performing treadmill training while receiving real-time visual biofeedback. The goal is to control minimum foot clearance (MFC) data within a specified range denoted by a red dot. This range is set as the (mean+SD)±(0.5*SD) of MFC, determining the upper and lower limits [6]. Adapted from [6], licensed under CC-BY 4.0 and the authors have made no changes to the original figure.

Biofeedback training provides individuals with real-time information about their body functions, enabling them to learn how to self-regulate and control those functions [6,8]. Our research group has pioneered treadmill-based biofeedback training by displaying the trajectory of a forefoot marker on a monitor positioned in front of the treadmill [6–8] to enhance MFC data through swing foot movement control (see Fig 1). It allows individuals to receive immediate visual feedback to manage MFC within a target range based on their swing foot movements. This training has been proven to be effective to improve MFC and recommended for stroke rehabilitation to prevent falls related to tripping [6,8].

Biofeedback-based training intervention has shown immediate, short-term, and long-term effects in stroke patients and healthy individuals. A positive, long-lasting impact of the task-specific visual biofeedback on equinovarus gait pattern among individuals with stroke was reported in [16]. A gait retraining program using real-time biofeedback successfully reduced knee hyperextension patterns in young women, with the improvements maintained for up to 8 months, suggesting potential long-term benefits of this intervention [17].

Studies in stroke and non-stroke individuals showed improved gait parameters after only a single biofeedback training session [18,19]. It was reported in [18] that neurologically unimpaired older adults were capable of improving anterior ground reaction force (AGRF) and gait speed. Likewise, it was found in [19] that seven young able-bodied individuals could enhance unilateral propulsion after just 11 minutes of walking at a self-selected pace on a dual-belt treadmill. In [20], an immediate increase in paretic AGRF, trailing limb angle (TLA), and step length was seen after just one session of visual biofeedback training. In [21], a study involved nine individuals with post-stroke hemiparesis who underwent three 6-minute training sessions on a treadmill with visual and auditory biofeedback to enhance paretic AGRF. Assessments were conducted before the training sessions and at 2, 15, and 30 minutes after training, revealing significant improvements.

The evidence of the immediate effect of biofeedback just after one training session, raises the possibility of differences in the ability to follow feedback in the initial session between individuals who experience immediate positive effects and those who do not. Additionally, the degree of adherence during the training session can have implications for the outcome of the assessment. If a patient shows a better adherence ability, it can be hypothesized that they are more likely to respond well to subsequent biofeedback training. Furthermore, the demonstrated adherence during the initial sessions can serve as an indicator of the assessment outcome conducted after multiple sessions. Despite prior studies highlighting the immediate and long-term impacts of biofeedback training intervention in stroke and non-stroke patients, there remains a research gap concerning whether a patient’s superior adherence to targeted biofeedback during the initial session could serve as an indicator of their ability to sustain this progress until the post-assessment. Furthermore, this adherence can be considered as a short-term effect observed during the real-time training, and it may provide insights into the potential long-term impact.

Our research analyses whether a patient’s Minimal Foot Clearance (MFC) data from the second real-time biofeedback session can predict improvement or lack thereof during post-assessment after multiple training sessions. We used data from the second session to minimize potential randomness resulting from patients adapting to the intervention system and to account for any carryover effects from the first session.

We employ four distance and two similarity metrics to evaluate the distinction between the second training session and baseline data [22]. We hypothesize that patients who successfully follow the targeted MFC will show less resemblance between their baseline and second training session data. These measures serve as features in our machine learning system, which aims to determine whether the effects of biofeedback training observed in the second session are sustained during the post-assessment phase. By analyzing adherence during early sessions, we seek to provide valuable insights into the likelihood of long-term improvement in stroke patients undergoing biofeedback training. Our key contributions to this work are:

- We propose a novel approach using cosine similarity (CS), correlation coefficient (CC) and cross-correlation distance (XCRD) measures to predict post-assessment improvements in stroke patients based on early biofeedback training data.
- Introduction of real-time adherence assessment metric (RAAM) to quantify improvements between consecutive training sessions, enabling more targeted interventions.
- We demonstrate that patients showing less similarity to their baseline during training sessions were likely to exhibit improved adherence to biofeedback and better outcomes.

Our work shows that patients who don’t show significant overall improvement may benefit from extended training periods, suggesting the potential for personalized rehabilitation strategies.

## 2 Related work

Existing research has predominantly concentrated on examining the linear statistical properties of biomechanical variables to explore lower-limb control characteristics analyzing MFC data.

Khandoker *et al*. [23] conducted a study where descriptive statistics, including mean, median, standard deviation (SD), 25th percentile (Q1), 75th percentile (Q3), and interquartile range (IQR), were computed to quantify the MFC series. They further introduced tone and entropy features derived from the percentage change in successive MFC points relative to the previous MFC point, known as the percentage index (PI). These tone and entropy features provided additional insights into the characteristics of the MFC data.

In another study, Khandoker *et al*. [24] recognized the complex patterns and nonstationary characteristics of the MFC time series. They utilized a wavelet-based multiscale exponent to capture correlations among the variances of wavelet coefficients at different scales. The MFC series was decomposed using Daubechies wavelets of order six and underwent eight decomposition levels. They analyzed the MFC time series across multiple scales and investigated the relationships between the series and various frequency components.

However, our approach focuses on utilizing distance and similarity metrics applied directly to the MFC series without any feature extraction or transformation. Additionally, we calculate similarity and distance metrics after extracting each of these features [23,24] from both the baseline and second training session MFC series.

## 3 Materials and method

### 3.1 Participants

This study included 15 patients over 18 years of age with at least six months after a single stroke (ischemic or hemorrhagic), with the ability to walk independently for 50 meters with or without a single point stick and the ability to provide informed consent were selected for biofeedback training [7]. Patients were excluded if they had ankle orthosis, any other medical condition that prevented them from walking on a treadmill, or any visual problem and a weight greater than 158 kg (weight limit problems of the harness) [7]. The participants were carefully informed, and their consent was secured to ensure informed participation. Informed written consent was obtained as per protocol and witnessed by the project staff member. The project staff (physiotherapist) explained the protocol and what participants needed to do, answering any questions before they signed up. Participants also received a copy of the project protocol prior to coming in for testing to read, understand, think about any questions, and share with their family or doctor.

The study (registered in the Australian and New Zealand Clinical Trials Registry - trial ACTRN12617000250336) was approved by the Human Research Ethics Committees of Victoria University, Australia, and Austin Hospital, Melbourne, Australia. The recruitment of the participants commenced on November 18, 2016, and was completed on March 22, 2023. Table 1 provides detailed information about the participants.

**Table 1:** The table summarizes the participants’ details, including the number of subjects, age, affected lower limb, and walking speed among 15 patients.

Class	Improved	Unimproved
No. of subjects	11	4
Age (years)	69.45±12.00	71±12.93
Female	3	1
Left affected	4	4
Walking speed (km/h)	1.96±1.50	2.27±0.25

### 3.2 Data collection

We employed a three-dimensional motion analysis system (Optotrak^®^, NDI, Canada) to capture kinematic data at 100 Hz. Following a standardized protocol [25], participants were outfitted with a cluster of three active markers, including one affixed to the big toe. The forefoot’s imaginary position was digitized using an active digitizing probe. To ensure safety and adherence to the protocol, all participants were secured by a safety harness and instructed to walk on a motorized treadmill at their self-selected walking speed for up to 10 minutes, with intervals of rest and breaks, as needed.

During subsequent biofeedback gait training sessions, the real-time sagittal trajectory of the big toe marker was displayed on a screen in front of the treadmill (see Fig 1). This display featured clear visuals of toe clearance and associated MFC events. The display also included a target range of MFC (calculated from baseline MFC data) depicted as a horizontal line on the screen [7]. Participants were then tasked with adjusting their MFC height to match the monitored ranges.

Patients underwent ten biofeedback training sessions, with faded biofeedback introduced after six initial sessions. Detailed information about the biofeedback training sessions is available in [7].

### 3.3 Assessment

Gait assessment evaluations were scheduled at the baseline and directly after the last training session (with a minimum interval of 20 minutes). After training, the participants were categorized as improved or unimproved. This categorization was determined based on the post-training MFC change from the baseline MFC data.

### 3.4 Computation of distance and similarity metrics

To evaluate the effectiveness of a training intervention, the baseline data serves as a reference point representing the measurements before any training occurred. The goal is typically to observe improvements in the post-training data compared to the baseline. Differences between the MFC series of the baseline and the second training session are computed by four *distance metrics*, and two *similarity metrics* [22,26]. Distance metrics quantify dissimilarity and focus on how far apart or different two points are, while similarity metrics measure resemblance and focus on how similar or related two vectors are. The range of distance metrics is typically from 0 to infinity, indicating higher values for more significant dissimilarity. Here, normalization is performed to make the distance metrics within the range of 0 to 1. Similarity metrics range from -1 to 1, with higher values indicating more remarkable similarity or robust relationships. We applied amplitude normalization to reduce sensor noises and eliminate amplitude-related variations caused by subjective physiological differences. Additionally, we perform zero-padding on the MFC series data, extending each series to 200 points before computing the four distance and similarity metrics mentioned below.

#### 3.4.1 Distance metrics.

Here, we have used four popular distance metrics: (i) Euclidean distance (ED), (ii) Manhattan distance (MD), (iii) Dynamic time warping distance (DTW) and (iv) Cross-correlation distance (XCRD).

- **Euclidean distance** measures the straight-line distance between two points, considering the magnitude and direction of differences. In this context, it measures the difference between baseline and second training session data by calculating the distance between corresponding MFC points. A smaller distance suggests a smaller difference, indicating similarity, while a larger distance signifies significant changes or variations between the two states. This insight helps objectively assess the impact of the training session. The normalized Euclidean distance between vectors **X** and **Y** of length or dimension *n* can be calculated as:
  $$ED=\frac{\sqrt{\sum_{i=1}^{n}(X_{i}-Y_{i}{)}^{2}}}{n}$$ (1)
- **Manhattan distance**, also known as the *L*1 distance or City Block distance, measures the total distance along the axes required to reach from one point to another, considering only absolute differences and ignoring specific relationships between dimensions. A smaller distance between the baseline and the second training session indicates higher similarity, while a larger distance signifies greater differences. This insight enables an objective assessment of the training session’s impact compared to the baseline.
  The normalized Manhattan distance between two vectors **X** and **Y** of dimension *n* can be computed as:
  $$MD=\frac{\sum_{i=1}^{n}|X_{i}-Y_{i}|}{n}$$ (2)
- **Dynamic time warping** measures diatance between two time series with different or varying speed or of different length [27,28]. It calculates a cost matrix *D*, where *D*(*i*,*j*) is the cumulative cost of aligning the first *i* elements of *X* with the first *j* elements of *Y*. It can be written as:
  $$D(i,j)=d(X_{i},Y_{j})+\min\left\{ \begin{array}{c} D(i-1,j)\\ D(i,j-1)\\ D(i-1,j-1) \end{array}\right.$$
- where, $d(X_{i},Y_{j})$ is the local cost between the two points *X*_*i*_ and *Y*_*j*_. Here, *i* and *j* are same as we have used zero-padded signal. We have calculated the normalized value by dividing the length of the signal, i.e, *n*.
- **Cross-correlation distance** provides a measure of dissimilarity between two time series by considering both immediate and delayed correlations [29,30]. A smaller distance between the baseline and the second training session indicates a greater similarity, while higher distances indicate greater differences.
  $$XCRD(X,Y)=\sqrt{\frac{1-XCR(X,Y,0{)}^{2}}{\sum_{k=1}^{\text{max\_lag}}(1-XCR(X,Y,k{)}^{2})}}$$
- where, *XCRD*(*X*,*Y*) is cross-correlation distance between *X* and *Y*, *XCR*(*X*,*Y*,0) is cross-correlation at lag 0, *XCR*(*X*,*Y*,*k*) is cross-correlation at lag *k*, max_lag is maximum lag.

#### 3.4.2 Similarity metrics.

Here, we have used two similarity metrics [31]: (i) Correlation coefficient (CC) and (ii) Cosine similarity (CS).

- **Correlation coefficient** quantifies the strength and direction of the linear relationship between variables. In this context, a significant linear relationship between the baseline signal and the signal during training indicates a minimal alteration in the overall pattern or trend. Conversely, a weak or near-zero correlation coefficient suggests a feeble or nonexistent linear relationship, indicating that the training effect may have introduced non-linear changes in the relationship between the variables, leading to inconsistent or disproportional modifications in the values of the two signals. It can be defined as:
  $$\text{CC}=\frac{\sum_{i=1}^{n}(X_{i}-\bar{X})(Y_{i}-\bar{Y})}{\sqrt{\sum_{i=1}^{n}(X_{i}-\bar{X}{)}^{2}\sum_{i=1}^{n}(Y_{i}-\bar{Y}{)}^{2}}}$$ (3)
- **Cosine similarity** quantifies the similarity between two vectors by measuring the cosine of the angle between them, providing insights into the overall similarity or dissimilarity between vectors, considering their orientations. In the case of the baseline and training data, cosine similarity can reveal how closely aligned or similar the two data sets are. A high cosine similarity indicates a strong alignment between the training data and the baseline, suggesting that the training has not resulted in significant deviations from the initial measurements. It can be defined between two vectors of dimensions *n*, denoted as *X* and *Y*, and can be calculated using the following formula:
  $$\text{CS}=\frac{\sum_{i=1}^{n}X_{i}Y_{i}}{\sqrt{\sum_{i=1}^{n}X_{i}^{2}}\sqrt{\sum_{i=1}^{n}Y_{i}^{2}}}$$ (4)

### 3.5 Predicting the improvement of MFC

To predict the improvement in MFC data from baseline treadmill training, we employ five classifiers [32]: Support Vector Machine (SVM), Random Forest (RF), AdaBoost, Ensemble Decision Tree (EDT), and Artificial Neural Network (ANN).

**Support Vector Machine (SVM)** is a supervised machine learning method based on the structural risk minimization principle [33]. It handles classification and regression by finding an optimal hyperplane to separate data points into distinct classes with maximum margin. The weight vector, bias, feature vectors, labels, Lagrange multipliers, and a kernel function determine the decision function. The decision function is given by:

$$f(x)=\text{sgn}\left(w.x+b\right)=\text{sgn}\left(\sum_{i=1}^{M}\left(\alpha_{i}y_{i}K(x_{i},x)+b\right)\right)$$ (5)

where *f*(*x*) represents the decision function, *w* is the weight vector perpendicular to the separating hyperplane, *b* serves as a bias determining the position of the hyperplane, *x*_*i*_ represents the *i*-th feature vector of dimension *d*, $y_{i}\in \{+1,-1\}$ is the label (target output) of *x*_*i*_, $\alpha_{i}$ is the Lagrange multiplier of the *i*-th data point, *K*(*x*_*i*_,*x*) is the kernel function, and *M* represents the number of *support vectors*—data points in the margin. The sgn(·) function returns the sign of the argument.

Kernels help separate classes by transforming data into higher dimensions when not separable in lower dimensions, offering options like polynomial and radial basis functions alongside linear kernels [33].

**Decision Tree** is a non-parametric supervised method for classification and regression [34], utilizing a tree-like structure for classification or prediction.

1. **Random Forest (RF)** uses multiple Decision Trees through *bagging*, training each tree on a random subset of the data and considering only a random subset of features at each split [35].
2. **AdaBoost** (Adaptive Boosting) is a boosting algorithm that sequentially combines weak learners (like shallow Decision Trees). It trains them on weighted data, emphasizing previously misclassified samples in subsequent iterations to enhance performance [36].
3. **Ensemble Decision Tree (EDT)** with *bagging* combines multiple Decision Trees trained on different bootstrap samples of the training data. This approach boosts stability and reduces variance by introducing randomness. The final prediction comes from merging individual tree predictions through majority voting or averaging methods [37].

**Artificial Neural Networks (ANNs)** consist of artificial neurons that mimic biological neurons of the human brain. They have interconnected layers, including an input layer receiving data and an output layer generating predictions. The output response is defined by a non-linear function applied to the weighted sum of hidden layer outputs. The output response is defined as:

$$o_{k}=f_{o}\left(\sum_{j=0}^{m}w_{k,j}h_{j}\right),$$ (6)

where *o*_*k*_ is the produced response of the *k*-th node of the output layer, *f*_*o*_ is the non-linear function at the output layer node, *m* is the number of nodes in the hidden layer, *w*_*k*,*j*_ is the weight connecting the *j*-th hidden node and the *k*-th output node, and *h*_0_ = 1 is the bias term.

ANNs are supervised classifiers where weights are set during training. Backpropagation and optimization methods adjust these weights between neurons to minimize output errors. Precise weight learning is crucial, with optimization algorithms fine-tuning weights through mathematical processes [38].

### 3.6 Model training and evaluation

Due to the imbalanced nature of our dataset, consisting of 4 samples in one class and 11 samples in the other, we have employed three approaches (i) 5-fold stratified cross-validation on the original dataset, (ii) the undersampling method, and (iii) the oversampling method.

In the 5-fold stratified cross-validation approach, the dataset is divided into five folds. The model is trained on four of these folds, while one fold is reserved for testing. This process is repeated five times, ensuring that each fold preserves the original class distribution. As a result, the training and testing sets maintain the same distribution as the original dataset. After completing the 5-fold cross-validation, we calculate the average performance metrics, which helps evaluate the model’s robustness and reliability across multiple iterations.

To further mitigate the impact of class imbalance on performance metrics, we have employed random undersampling [39,40] and Synthetic Minority Over-sampling Technique (SMOTE)-based oversampling techniques [41]. For random undersampling, we have randomly selected three samples from each class for training, with the rest being used for testing. This random selection has been repeated 200 times to obtain average performance metrics. Additionally, we have applied SMOTE [41–43] (utilizing two nearest neighbors) to the training folds during each iteration of 5-fold stratified cross-validation and calculated the average performance metrics.

To ensure generalization, performance metrics such as accuracy (ACC), sensitivity (SENS), specificity (SPEC), and F1 score (F1) are calculated [44]. These three approaches evaluate the model’s performance while accounting for variations between subjects and ensuring its ability to generalize to unseen data.

### 3.7 Feature selection based on ranking and majority voting

Selecting relevant and non-redundant features is crucial for the classifier’s performance [25]. We used the minimum redundancy maximum relevance (mRMR) feature selection method to rank four features based on their relevance to the target variable and the redundancy they introduce [45]. The mRMR method aims to maximize the relevance of selected features to the target variable while minimizing redundancy among them. Due to the limited sample size, separately we stratified the data into five and three folds for feature selection. Employing a majority voting approach across the folds, we selected features that consistently ranked at the top in most of the folds.

### 3.8 Real-time adherence assessment metric (RAAM)

This section introduces our proposed *real-time adherence assessment metric (RAAM)* for measuring adherence between consecutive training sessions.

The Gait Profile Score (GPS) is a single index measure that summarizes the overall deviation of kinematic gait data relative to normative data [46,47]. Similar to the notion of GPS variation computation in [47], we measure improvement in adherence from one training session to another. We used 200 points (i.e., MFCs) corresponding to 200 strides. Instead of a gait cycle and nine measurements in [47], we use one MFC value measurement across 200 gait strides. We denote the gait session *E*_*p*,*d*_ of a patient *p* at datetime *d* as

$$E_{p,d}=[\begin{array}{c} C_{E_{p,d}}^{1},C_{E_{p,d}}^{2},\ldots ,C_{E_{p,d}}^{K} \end{array}]$$ (7)

where, $C_{E_{p,d}}^{k}$ is the *k*-th gait cycle of a gait session and *K* is the total number of gait cycles. We modify this notation in our case with a vector of *t* = 200 lines representing 200 strides instead of a single stride and *n* = 1 column, representing the MFC values as:

$$C_{E_{p,d}}^{K{=}_{\left[1:200\right]}}=[\begin{array}{c} c_{t,n} \end{array}]=[\begin{array}{c} c_{1,1}\\ c_{2,1}\\ \vdots \\ c_{200,1} \end{array}]$$ (8)

Instead of using normative data as a reference point, we have used each patient’s baseline. This baseline serves as the starting point from which we assess improvement. Then, we will use *m*^*th*^ similarity and distance metrics (represented as *SIMDIST*) to compute the MFC Variation Score (MVS) as

$$MVS_{m}=\text{SIMDIST}(c_{t,n},c_{\text{ref}\left(t,n\right)})$$ (9)

Then, we use Eq (9) to compute MFC-based GPS as

$$GPS_{MFC}=\sqrt{\frac{1}{N}\sum_{m=1}^{N}MVS_{m}^{2}}$$ (10)

where, *N* is the number of similarity and distance measures.

We hypothesize that choosing one or multiple similarity and distance measures that effectively differentiate between baseline and training sessions would be more advantageous in *GPS*_*MFC*_ computation.

For (*i* + 1)^*th*^ training session, *GPS*_*MFC*_(*i* + 1) variation from the previous session can be computed using:

$$\Delta GPS_{MFC}(i+1)=GPS_{MFC}(i+1)-GPS_{MFC}(i)$$ (11)

We get 9 $\Delta GPS_{MFC}$ as we have 10 training sessions. We need to be cautious in using these metrics as either similarity or distance measures during the calculation of $MVS_{m}$. When we exclusively calculate similarity, if $\Delta GPS_{MFC}(i\;+\;1)$ is negative, it indicates an enhancement in the patient’s adherence ability from *i*^*th*^ to $(i\;+\;1){\;}^{th}$ session. In contrast, if positive, it signifies a decline from the previous session.

If there is a distance metric present in the feature set, it is better to convert other similarity values into distance values for calculating $MVS_{m}$. Since CC and CS range from 0 to 1, we can obtain distance values by subtracting them from 1, i.e., 1–*CC* or 1–*CS*. When using these distance measures, a negative value for $\Delta GPS_{MFC}(i\;+\;1)$ indicates a decrease in the patient’s adherence ability from the *i*^*th*^ to the $(i\;+\;1){\;}^{th}$ session. Conversely, a positive value signifies an improvement from the previous session.

## 4 Results

We structure our findings and analytical discussion into five distinct subsections, each addressing a crucial aspect of our research: (a) Analysis of selected features, (b) Performance comparison of selected feature subset in different classifiers, (c) Performance of similarity measures evaluated from alternative MFC features, (d) Adherence indication via similarity measures and (e) Inter-session improvement assessment.

### 4.1 Analysis of selected features

**Fig 2:**
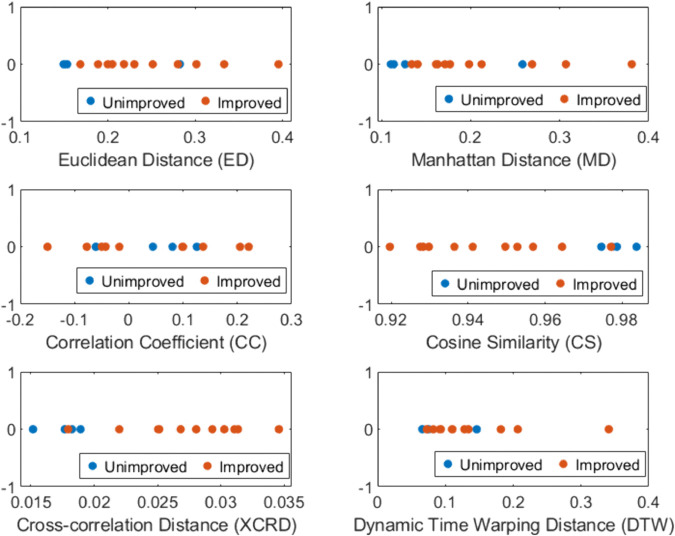
This figure shows the variations in features across the two classes, utilizing six metrics: Euclidean distance (ED), Manhattan distance (MD), correlation coefficient (CC), cosine similarity (CS), cross-correlation distance (XCRD) and dynamic time warping distance (DTW). It highlights that distance metrics (ED, MD, XCRD and DTW) are comparatively lower between the baseline and the second training session for unimproved participants than for improved participants. Low CC value suggests training impact with a weak linear relationship (i.e., mean shift) between baseline and second training MFC. CS indicates higher similarity between baseline and second training for unimproved patients compared to improved ones.

Fig 2 illustrates six extracted features that capture the distance and similarity between the MFC series of the baseline and second training session. The figure shows that the distance metrics (Euclidean and Manhattan) have lower values between the baseline and the second training session for three out of four unimproved patients compared to the improved patients. In the case of cross-correlation distance and dynamic time warping distance, the unimproved class exhibits a lower distance compared to the improved class. However, for DTW, the difference between the two classes is not very pronounced. However, interpreting the correlation coefficient between the MFC series is more complex. The low correlation coefficient values (regardless of their positive or negative nature) reveal that the training has an impact, resulting in a weak linear relationship between the baseline MFC and the second training MFC. Regarding cosine similarity, the figure also indicates that the unimproved class patients exhibit higher similarity between the baseline and the second training session compared to the improved class.

After applying the mRMR-based feature selection, CS, XCRD, CC, and DTW features are consistently selected as the top-ranked variables in most cases, while ED and MD have ranked the lowest. The performance of the selected feature subsets in the SVM classifier, is presented in Tables 2, 3 and 4. To determine the best-performing feature set, we focused on features that consistently delivered the best F1-scores across the majority of scenarios. The decision to use the F1-score was based on its robustness in scenarios with data imbalance [48,49]. We have analyzed all three tables to determine which combination of features performs best in most cases. In Table 2, the RBF kernel yields the best outcomes for the combinations CC + CS, CC + XCRD, and CC + XCRD + CS. Table 3 indicates that the combination CS + XCRD achieves the best results across nearly all kernels. Table 4 highlights that CC + CS, CS + XCRD, and CC + CS + XCRD mostly excel in the RBF kernel.

**Table 2:** Performance comparison of the selected features with the SVM Classifier utilizing five-fold stratified cross validation. Best performance for a C value among the kernel is presented. Highest performances are highlighted, along with the feature set and kernel that achieved it. Evaluation Metrics: Accuracy (ACC), Sensitivity (SENS), Specificity (SPEC), and F1-score (F1). Similarity Measures: cs: Cosine Similarity, cc: Correlation Coefficient, md: Manhattan Distance, ed: Euclidean Distance, xcrd: Cross-correlation Distance, dtw: Dynamic Time Warping Distance.

	Linear					RBF				Polynomial			
Feature	C	Acc	Sens	Spec	F1	Acc	Sens	Spec	F1	Acc	Sens	Spec	F1
cc+cs+dtw+xcrd+md+ed	1000	80.00	80.00	75.00	82.67	73.33	90.00	25.00	81.33	73.33	100.00	0.00	84.00
cc+cs+dtw+xcrd+md	1000	80.00	80.00	75.00	82.67	66.67	80.00	25.00	75.33	73.33	100.00	0.00	84.00
cc+cs+dtw+xcrd	10000	86.67	80.00	100.00	86.67	93.33	90.00	100.00	93.33	86.67	80.00	100.00	86.67
cc+dtw+xcrd	10000	80.00	80.00	75.00	82.67	66.67	76.67	50.00	75.33	73.33	100.00	0.00	84.00
**cc+cs+xcrd**	10000	93.33	90.00	100.00	93.33	100.00	100.00	100.00	100.00	93.33	90.00	100.00	93.33
cc+cs+dtw	10000	86.67	80.00	100.00	86.67	93.33	90.00	100.00	93.33	80.00	80.00	75.00	83.33
dtw+xcrd	10000	86.67	90.00	75.00	89.33	86.67	90.00	75.00	89.33	73.33	100.00	0.00	84.00
cs+xcrd	10000	93.33	90.00	100.00	93.33	93.33	90.00	100.00	93.33	93.33	90.00	100.00	93.33
cs+dtw	10000	86.67	80.00	80.00	86.67	93.33	90.00	80.00	93.33	93.33	90.00	80.00	93.33
**cc+xcrd**	10000	80.00	80.00	75.00	82.67	100.00	100.00	100.00	100.00	73.33	100.00	0.00	84.00
cc+dtw	0.1	73.33	100.00	0.00	84.00	73.33	100.00	0.00	84.00	73.33	100.00	0.00	84.00
**cc+cs**	1000	80.00	80.00	75.00	82.67	100.00	100.00	100.00	100.00	93.33	90.00	100.00	93.33

**Table 3:** Performance comparison of the selected features with the SVM Classifier utilizing undersampling method. Best performance for a C value among the kernel is presented. Highest performances are highlighted, along with the feature set and kernel that achieved it. Evaluation Metrics: Accuracy (ACC), Sensitivity (SENS), Specificity (SPEC), and F1-score (F1). Similarity Measures: cs: Cosine Similarity, cc: Correlation Coefficient, md: Manhattan Distance, ed: Euclidean Distance, xcrd: Cross-correlation Distance, dtw: Dynamic Time Warping Distance.

	Linear					RBF				Polynomial			
Feature	C	Acc	Sens	Spec	F1	Acc	Sens	Spec	F1	Acc	Sens	Spec	F1
cc+cs+dtw+xcrd+md+ed	10000	63.67	63.50	64.82	72.86	63.06	62.81	64.82	72.06	58.61	58.38	60.30	69.00
cc+cs+dtw+xcrd+md	10000	63.33	63.13	64.82	72.80	63.33	63.13	64.82	72.78	63.22	63.38	61.81	73.23
cc+cs+dtw+xcrd	1000	62.56	60.75	76.88	71.78	57.39	55.75	70.35	67.36	50.06	47.00	74.37	60.02
cc+dtw+xcrd	10000	80.89	80.81	81.41	87.58	81.11	81.06	81.41	87.67	81.83	80.31	93.97	88.20
cc+cs+xcrd	10000	71.67	69.44	89.45	79.43	72.00	69.50	91.96	79.64	74.28	72.00	92.46	81.81
cc+cs+dtw	10000	62.33	60.69	75.38	71.26	64.00	62.63	74.87	72.67	68.56	66.69	83.42	77.24
dtw+xcrd	10000	79.50	80.06	86.62	86.62	78.61	79.06	86.00	86.00	33.17	27.13	36.41	36.41
**cs+xcrd**	10000	86.22	84.50	100.00	91.26	86.22	84.50	100.00	91.26	86.39	84.69	100.00	91.40
cs+dtw	10000	80.50	80.38	81.41	87.32	81.11	81.06	81.41	87.68	84.56	83.38	93.97	89.96
cc+xcrd	10000	70.11	67.88	87.94	78.20	69.06	67.50	81.41	77.02	25.44	18.63	80.40	26.97
cc+dtw	10000	42.00	41.19	48.24	51.67	40.50	39.38	49.25	50.51	22.89	14.13	92.96	21.55
cc+cs	10000	71.61	69.38	89.45	79.38	71.72	69.44	89.95	79.39	73.44	71.00	92.96	80.94

**Table 4:** Performance comparison of the selected features with the SVM Classifier utilizing SMOTE method. Best performance for a C value among the kernel is presented. Highest performances are highlighted, along with the feature set and kernel that achieved it. Evaluation Metrics: Accuracy (ACC), Sensitivity (SENS), Specificity (SPEC), and F1-score (F1). Similarity Measures: cs: Cosine Similarity, cc: Correlation Coefficient, md: Manhattan Distance, ed: Euclidean Distance, xcrd: Cross-correlation Distance, dtw: Dynamic Time Warping Distance.

	Linear					RBF				Polynomial			
Feature	C	Acc	Sens	Spec	F1	Acc	Sens	Spec	F1	Acc	Sens	Spec	F1
cc+cs+dtw+xcrd+md+ed	1000	80.00	80.00	75.00	82.67	73.33	73.33	75.00	78.67	60.00	60.00	75.00	62.67
cc+cs+dtw+xcrd+md	1000	80.00	80.00	75.00	82.67	73.33	73.33	75.00	78.67	60.00	60.00	75.00	62.67
cc+cs+dtw+xcrd	10000	86.67	80.00	100.00	86.67	93.33	90.00	100.00	93.33	86.67	80.00	100.00	86.67
cc+dtw+xcrd	10000	66.67	63.33	75.00	72.00	73.33	76.67	75.00	79.33	33.33	10.00	100.00	13.33
**cc+cs+xcrd**	10000	86.67	80.00	100.00	86.67	100.00	100.00	100.00	100.00	86.67	80.00	100.00	86.67
cc+cs+dtw	10000	86.67	80.00	100.00	86.67	93.33	90.00	100.00	93.33	86.67	80.00	100.00	86.67
dtw+xcrd	10000	80.00	80.00	75.00	82.67	80.00	80.00	75.00	82.67	40.00	20.00	100.00	20.00
**cs+xcrd**	10000	86.67	80.00	100.00	86.67	100.00	100.00	100.00	100.00	100.00	100.00	100.00	100.00
cs+dtw	10000	86.67	80.00	100.00	86.67	93.33	90.00	100.00	93.33	93.33	90.00	100.00	93.33
cc+xcrd	10000	86.67	80.00	100.00	86.67	93.33	90.00	100.00	93.33	40.00	20.00	100.00	26.67
cc+dtw	1000	53.33	60.00	50.00	59.33	53.33	60.00	50.00	59.33	33.33	10.00	100.00	13.33
**cc+cs**	10000	86.67	80.00	100.00	86.67	100.00	100.00	100.00	100.00	100.00	100.00	100.00	100.00

**Fig 3:**
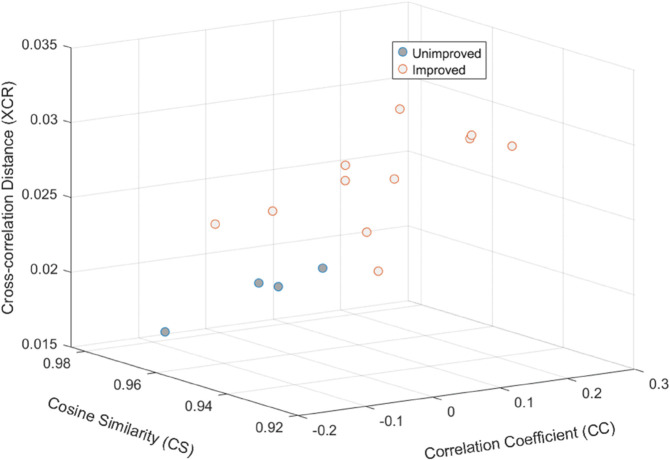
This scatter plot shows the discriminatory capability of features to separate unimproved and improved classes using the CS,CC and XCRD-based feature selection and majority voting across five folds.

Notably, the features based on CS, CC, and XCRD have proven to be better performers across the three classification scenarios, surpassing others and achieving 100% correct predictions in some instances. As no single set of features consistently outperforms in all three scenarios, we opted to include each feature that enhances performance in at least two of the scenarios. We want to ensure that we do not overlook any feature that provides value in one of the scenarios. This highlights the significance of each feature, contributing to a more robust feature set. Based on these findings, we have determined that CC + CS + XCRD is the optimal combination. Fig 3 illustrates that the combined feature of CC, CS and XCRD exhibits superior separability between the improved and unimproved classes compared to individual measures in Fig 2.

### 4.2 Performance comparison of selected feature subset in different classifiers

Table 5 summarizes the performance of five machine learning models after fine-tuning their respective hyperparameters. The models evaluated are Support Vector Machine (SVM) (https://www.csie.ntu.edu.tw/cjlin/libsvm/), Random Forest (RF), Ensemble Decision Trees (EDT), AdaBoost, and Artificial Neural Network (ANN).

**Table 5:** Classification performance metrics of selected feature (CC + CS + XCRD) among five different classifiers utilizing five-fold cross validation. Metrics: accuracy (ACC), sensitivity (SENS), specificity (SPEC), and F1-score (F1).

Classifier	Acc	Spec	Sens	F1
SVM	**100**	**100**	**100**	**100**
RF	93.33	100.00	90.00	93.33
Adaboost	93.33	75.00	100.00	96.00
EDT	93.33	100.00	90.00	93.33
ANN	**100**	**100**	**100**	**100**

For the SVM, we explored three kernels (Linear, RBF, and Polynomial of degree 3) and varied the c value from 0.1 to 10000. In the case of RF and EDT, we experimented with the minimum leaf size (1, 5, and 10) and the number of trees (randomly chosen between 5 and 100). The AdaBoost classifier was fine-tuned by adjusting the learning rate (0.001, 0.01, and 0.1) and the number of weak learners (15, 20, 25, and 30).

For the ANN, we varied the learning rate (0.1, 0.01, and 0.001) and the number of hidden nodes (10, 20, and 50) within a single hidden layer (i.e., Multilayer perceptron(MLP)). Additional hyperparameter optimization for the ANN included:

- Optimization functions: Gradient Descent Backpropagation, Fletcher-Reeves Conjugate Gradient Descent, and Polak-Ribiére Conjugate Gradient Descent
- Transfer functions: tansigmoid and log-sigmoid
- A fixed number of epochs (1000)
- A ridge regularization value of 0.01

Based on the results, both SVM and ANN achieved the highest performance 100% across all performance metrics when utilizing selected cosine similarity, correlation coefficient and cross-correlation distance features.

### 4.3 Performance of similarity measures evaluated from alternative MFC features

We employed three distinct approaches to analyze the baseline and second training data: wavelet-based multi-exponent features [24], statistical and tone-entropy features [23], and raw MFC series analysis. Also, we combined all three features to capture complimentary information of each of the feature. For the wavelet-based approach, we conducted wavelet decomposition at eight levels on both the baseline and second training MFC series, extracting six features from each decomposition. We then calculated the CC, CS and XCRD between the baseline and second training wavelet features to assess their similarity.

Our statistical and tone-entropy analysis involved computing various descriptive statistics for MFC series, including mean, median, standard deviation, quartiles, and interquartile range. Additionally, we evaluated tone and entropy estimates from the MFC Percentage Index (PI) series. We calculated CC, CS and XCRD between the baseline and second training statistical-tone-entropy features to quantify their similarity. For the raw MFC series analysis, we calculated CC, CS and XCRD between the baseline and second training MFC series without any feature extraction.

**Fig 4:**
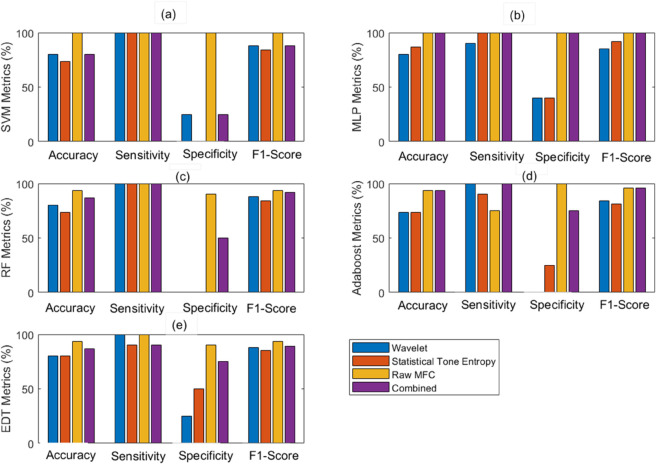
The figure displays the performance of five classifiers: (a) SVM, (b) MLP, (c) RF, (d) AdaBoost, and (e) EDT. The evaluation involves the utilization of correlation coefficient (CC), cosine similarity (CS) and cross-correlation distance (XCRD) measures for Wavelet features [24], Statistical Tone Entropy (statTE) features [23], and the raw MFC data points. The raw MFC data shows better performance compared to wavelet, statistical-tone-entropy and combined features.

To evaluate the performance of these different approaches, we employed five classifiers. We compared their performance using similarity measures derived from each method: wavelet-based, statistical-tone-entropy-based, raw MFC series, and all three features together (i.e., combined). Our results showed in Fig 4 that the raw analysis of MFC series, which used similarity measures computed directly from the MFC values without feature extraction, consistently outperformed both wavelet-based methods, statistical tone entropy, and combined features. This superior performance was evident across all metrics except for sensitivity in the Adaboost classifier.

These findings demonstrate the effectiveness of using these measures (CC, CS and XCRD) computed directly from MFC values than those derived from extracted features. The results suggest that, in this context, the raw data contains valuable information that may be partially lost in the feature extraction process. It is observed that, despite combining all three features, this combination fails to capture any additional complementary information that would enhance performance compared to the raw one. However, the utility of the feature-based approaches should not be discounted, as they offer insights into specific aspects of the data that are not immediately apparent in the raw series.

### 4.4 Adherence indication via similarity and distance measures

**Fig 5:**
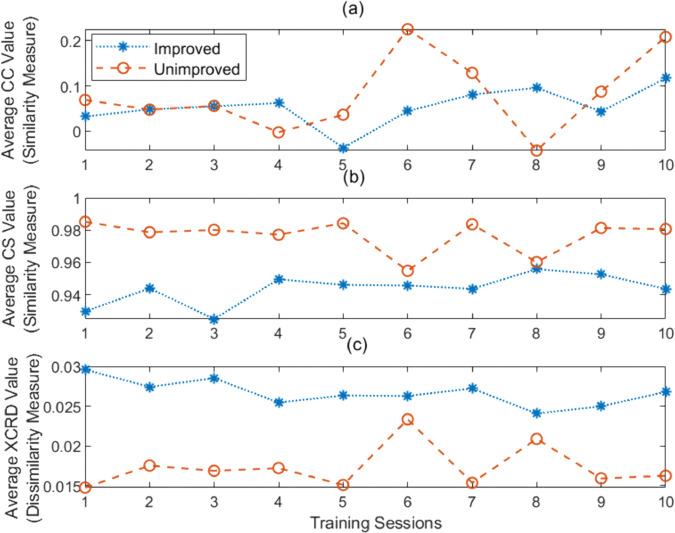
The figure shows the average (a) CC (b) CS and (c) XCRD-based measures over 10 training sessions for all patients in two groups, demonstrating the trend of the impact of targeted biofeedback between the baseline and each training session. CC and CS represent similarity measures. A low CC suggests a weak linear relationship between the baseline and the training session, while a high value indicates a strong linear relationship. A low CS value indicates that the subjects show less similarity to the baseline in that training session, while a high value suggests greater similarity. In contrast, the XCRD-based measure indicates dissimilarity: a high value in a particular training session means the subjects are less similar to the baseline condition, whereas a low value indicates a higher similarity. In (a), the CC-based measure indicates that the biofeedback has resulted in a weak linear relationship between the baseline and the training sessions. On the other hand in (b), the CS-based measure and in (c) XCRD-based measure clearly illustrate that when a patient’s training data is more similar to the baseline, they are less likely to show improvement during the post-assessment, and vice versa.

Fig 5 displays the average values of CC (Fig 5-(a)), CS (Fig 5-(b)) and XCRD (Fig 5-(c)) -based measures across all the 10 training sessions for both improved and unimproved stroke patients.

**Correlation Coefficient (CC) Analysis:** The CC-based measure indicates that a lower correlation value might portray the impact of biofeedback on the MFC values during training sessions, potentially causing them to shift in a weakly linear manner compared to the baseline. This suggests that the mean value of MFC during the training session has likely deviated from the mean value of the baseline MFC [26] for both classes of patients.

**Cosine Similarity (CS) Analysis:** The CS-based measure reveals an interesting pattern across training sessions. Patients who showed improvement in the post-assessment phase consistently exhibited less similarity to the baseline compared to those who did not improve. This difference in similarity scores between improved and unimproved patients is observed for each training session.

**Cross-correlation Distance (XCRD) Analysis:** The XCRD-based measure reveals that those who showed improvement in the post-assessment phase consistently exhibited more distance to the baseline compared to those who did not. This difference in distances between improved and unimproved is observed for all the training sessions.

### 4.5 Inter-session improvement assessment

Our investigation revealed that measures based on correlation coefficient (CC), cosine similarity (CS) and cross-correlation distance (XCRD) performed better in predicting improvements using only the second training dataset. In accordance with the methodology outlined in Sect 3.8 to perform inter-session improvement or decrement assessment, we conducted a statistical significance test of CC, CS and XCRD values in the improved and unimproved class categories. This analysis informed our decision on which similarity measure to utilize for calculating the $\Delta GPS_{MFC}$.

Using a non-parametric Mann-Whitney U test, we found that CS and XCRD values were statistically significant (p<0.05) for all training sessions except the 4th, 6th and 8th. This suggests that the assessment is reliable for most sessions. It has been observed that this exception occurred because one subject in the 6th training session and another subject in the 8th training session both from the unimproved class exhibited less similarity with the baseline. This may suggest that these patients made a greater effort to adhere to the target more effectively during those training sessions. Additionally, some exhibit a higher similarity to the baseline during some training session while being in the improved class.

**Fig 6:**
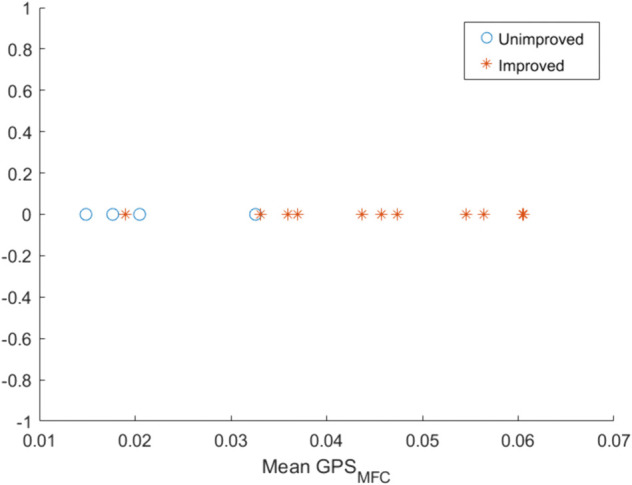
Figure represents mean of the ${GPS}_{MFC}$ values across the 10 training sessions for all the participants. It is observed that the mean *GPS*_*MFC*_ is higher (i.e., dissimilar to baseline ) in most of the cases of improved participants compared to unimproved ones.

**Fig 7:**
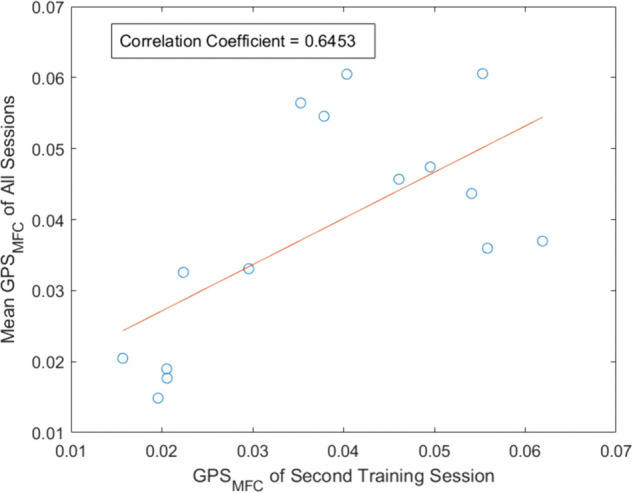
Figure represents correlation between ${GPS}_{MFC}$ of second training session and mean of the ${GPS}_{MFC}$ values across the 10 training sessions for all the participants. The mean *GPS*_*MFC*_ of the second training session shows a correlation coefficient of 0.6453 with the mean *GPS*_*MFC*_. This indicates a moderate to strong relationship between the *GPS*_*MFC*_ of second training session and the mean *GPS*_*MFC*_ across all sessions [50].

Fig 6 represents the mean value of *GPS*_*MFC*_ in all available training sessions of individual participants. It is observed that in most cases the mean *GPS*_*MFC*_ is lower for unimproved participants and higher for improved participants. Fig 7 illustrates the relationship between the *GPS*_*MFC*_ of the second training session and the mean *GPS*_*MFC*_ of all the training sessions. The correlation coefficient between the *GPS*_*MFC*_ value of the second training session and the mean *GPS*_*MFC*_ is 0.6453, indicating a moderate to strong correlation [50].

**Fig 8:**
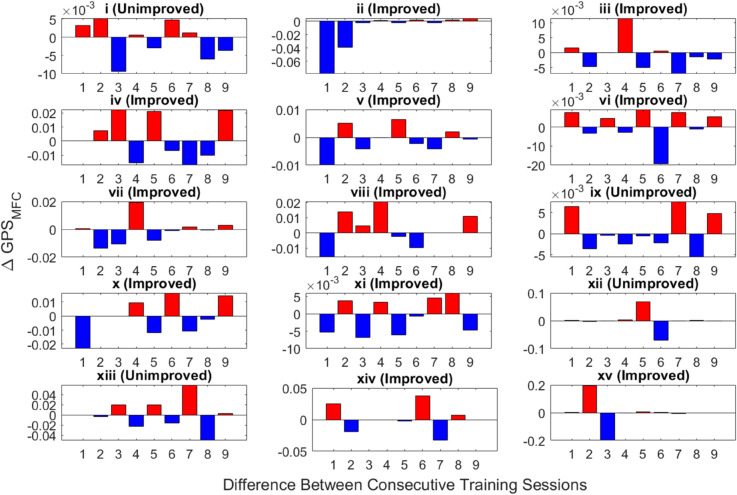
Illustration of improvement or decline of 15 stroke patients (i.e., (i)-(xv) ) between consecutive training sessions using the proposed $\Delta {GPS}_{MFC}$, with CS and XCRD employed as the dissimilarity measure from the baseline. From the 10 training sessions, nine values are computed. The first value signifies the difference in adherence capability between the second and first sessions, the second value represents the difference in adherence capability between the third and second sessions, and so forth up to the tenth session. The negative values of $\Delta GPS_{MFC}$ (represented in blue) computed across training sessions can be used to predict potential decrement in following the biofeedback, whereas positive values (represented in red) indicate a increment from the previous session for a specific patient.

Fig 8 illustrates the improvement or decline in adherence ability to feedback between consecutive training sessions using the measurement $\Delta GPS_{MFC}$, with CS and XCRD as dissimilarity measures. In Fig 8, the x-axis ranges from 1 to 9. The value *y* for *x* = 1 represents the difference in *GPS*_*MFC*_ between the second and first sessions, and similarly up to the value *y* for *x* = 9 corresponds to the difference in *GPS*_*MFC*_ between the tenth and ninth sessions. In some areas of Fig 8, $\Delta GPS_{MFC}$ is shown as zero. This is due to data loss during collection; although the participant completed those specific sessions, technical issues prevented the data from being captured. All calculations were made with this consideration including mean *GPS*_*MFC*_ and $\Delta GPS_{MFC}$. Across the training sessions, we use dissimilarity measure to compute *GPS*_*MFC*_, we observed (in Fig 8) (1) instances of improvement between consecutive sessions (indicated by positive values) and (2) cases where improvement was not evident (represented by negative values indicating decrement).

Notably, among stroke patients who did not show significant improvement during post-assessment (Fig 8 (i), (ix), (xii), and (xiii)), some displayed progress in some instances during consecutive training sessions. This suggests that (1) the observed differences may have been insufficient to demonstrate significant improvement during post-assessment, and (2) additional training sessions could potentially lead to significant enhancements within this group.

## 5 Discussion

The amount of adherence to real-time targeted biofeedback can be viewed as a short-term effect in stroke patients. It can serve as an indicator of their ability to follow the biofeedback. Patients who initially demonstrate a superior ability to follow the feedback compared to others might exhibit greater flexibility in consistently adhering to the targeted feedback.

This study hypothesized that the patients who can follow the biofeedback in the initial session might improve in the post-assessment. As we use the baseline MFC series as the reference point, it is natural that the patients with less similarity or specific difference from baseline follow the biofeedback better than others. We have used MFC data from the second training session instead of first session to minimize potential randomness caused by patients adapting to the intervention system and to avoid capturing erroneous data. By predicting outcomes during the post-training assessment using data from the second session, this approach reduces resource costs and saves time. We have used four distance metrics (ED, MD, XCRD and DTW) and two similarity metrics (CC and CS) to measure the distance and similarity between the second training session MFC series and baseline MFC series.

Based on Fig 2, three unimproved subjects exhibit lower distance values for ED and MD. This aligns with our assumption that unimproved individuals would have minimal deviation from the baseline. In case of XCRD, it is also seen that most unimproved subjects exhibit lower distance values. In case of DTW, although most unimproved subjects show lower distance values, the difference between unimproved and improved is not that prominent. In the case of the CC-based measure, it is observed that following feedback or adherence to it might cause a shift in the mean value of the MFC series during training. Consequently, this shift leads to a weak relationship with the baseline. When considering CS, all four unimproved patients demonstrated higher similarity with the baseline during the second training session.

Our mRMR-based feature selection revealed that cosine similarity (CS), cross-correlation distance (XCRD), correlation coefficient (CC) and dynamic time warping distance (DTW) are more effective metrics than Euclidean Distance (ED) and Manhattan Distance (MD) for comparing MFC series. Fig 3 demonstrates that combining CC, CS and XCRD provides better separation compared to using each metric individually (as shown in Fig 2).

CC measures the linear relationship between two series, indicating how closely their overall trends align. XCRD measures how well two series correlate with each other when one is shifted or lagged relative to the other. Conversely, CS assesses the similarity in shape and direction, regardless of magnitude differences. Focusing on these metrics, our analysis emphasizes capturing underlying patterns in the MFC series rather than just point-by-point differences.

In contrast, ED and MD primarily measure absolute differences between data points, potentially missing overall trend similarities. In the case of DTW, the separation between the two classes is not that prominent (refer to Fig 2). Therefore, CC, CS and XCRD appear more suitable for evaluating differences between the baseline and second training session MFC series, as they prioritize trend, shape and direction analysis as a critical characteristic.

The results presented in Table 5 demonstrate the superior performance of the CC, XCRD and CS-based feature, which achieved 100% accuracy in prediction. This exceptional performance highlights the feature’s discriminative solid power and ability to capture the essential characteristics required for accurate classification effectively. However, it is seen that adding MD,ED and DTW-based measures to the feature set led to a decline in performance. This suggests that these additional measures may be redundant to the classification task, as supported by mRMR feature selection.

Further analysis in Table 5 reveals that the CS-CC-XCRD-based feature performs best with SVM and ANN classifiers which might be due to the complex and non-linear nature of the data. Moreover, the CS-CC-XCRD-based features derived directly from the MFC series showed superior performance compared to those calculated from wavelet MFC and stat-tone-entropy MFC features. This observation implies that the derived features might be missing crucial information present in the untransformed MFC series. Although combining the proposed MFC, wavelet MFC, and stat-tone-entropy MFC features improves performance compared to using wavelet MFC or stat-tone-entropy MFC features individually in some classifiers, likely due to the capture of complementary information, it does not exceed the performance of the proposed method.

Upon examining the average CC, CS and XCRD values across all patients in both groups during each training session in Fig 5, the CC-based measure reveals that the shifting of the mean value may be responsible for the weak linear relationship between the baseline and the training session, which can be attributed to the effect of adherence during the real-time training session. Conversely, the CS and XCRD-based measure suggests that patients who demonstrated less similarity to the baseline during the training sessions were more likely to exhibit stronger adherence to biofeedback. In contrast, patients who did not show improvement showed higher CS measure or lower values of the XCRD measure, indicating a greater resemblance to baseline.

As illustrated in Fig 7, *GPS*_*MFC*_ of second training session and mean *GPS*_*MFC*_ has moderate to strong correlation [50]. Already, it is seen that the CS and XCRD values are statistically significant in differentiating progressing and nonprogressing groups during the second training session (refer to Sect 4.5). The correlation analysis, therefore, supports that improvement predictions using the second-session training data were reliable.

As illustrated in Fig 8, both the improved and unimproved patient groups occasionally showed better adherence (i.e., positive values of $\Delta GPS_{MFC}$) from one training session to the next, while at other times they did not (i.e., negative values of $\Delta GPS_{MFC}$). It is important to note that these data reflect real-time gait training in which participants are actively trying to follow the target foot trajectory feedback, rather than assessments conducted after the training session. It is also important to note that the total improvement with training reflects the accumulated effect of all 10 training sessions.

The key insight is that the average patterns of adherence indicators reveal that participants who improved with training utilized the visual display feedback more effectively than patients who were not predicted to show significant improvements to foot trajectory control, i.e., poorer adherence (refer to Fig 5). Consequently, when examining the mean *GPS*_*MFC*_ across all sessions, based on dissimilarity, in most cases the improved group demonstrated a higher mean *GPS*_*MFC*_ than the unimproved group (refer to Fig 6). In gait rehabilitation attention efforts should, therefore, be directed toward increasing $\Delta GPS_{MFC}$, indicating that the participant is employing feedback more effectively than in the previous session.

## 6 Conclusion

The objective of this study is to assess (i) if progress is noticeable following the initial training session of biofeedback-based treadmill training and (ii) if measurements between subsequent sessions can assist in guiding interventions for patients who are not progressing. Our findings demonstrate that both CS, CC and XCRD-based measures accurately predict improvements in post-assessment outcomes based on the baseline Minimum Foot Clearance (MFC) and second training MFC.

These similarity measures serve as valuable adherence indicators during training sessions, revealing short-term real-time effects of biofeedback that may influence long-term outcomes observed in post-assessment. Our results suggest that adherence to biofeedback leads to a mean shift, resulting in a weak linear relationship between the training session and the baseline. Notably, patients showing improvement during post-assessment displayed lower cosine similarity and higher cross-correlation distance between their baseline and training sessions than those without improvement. Interestingly, proposed adherence assessment metrics show that even patients in the unimproved group exhibit positive responses in certain training sessions when assessing progress or decline in each training session.

This study leads to two key conclusions that could serve as important metrics for guiding rehabilitation towards improved outcomes. First, whether a patient will show improvement after several sessions is reflected in their adherence to feedback during early training sessions. Second, if this adherence-based discrepancy between who will improve and who will not during post-training assessment is evident in the initial session, it should be there in the later sessions, and it can be used as a guide how biofeedback is influencing subsequent sessions. Therapists can compare a patient’s adherence profile to previous sessions; if patients are adhering to feedback more effectively than before, they may be on the right path towards improvement during post-assessment.

It is also important that adherence ability to feedback during any particular training session can be impacted due to internal or external factors for any particular patient. Therefore, characteristics of multiple training sessions might be necessary to determine whether a patient’s adherence to feedback reflects a genuine pattern toward future outcome during post-training assessments.

Future studies could explore the correlation between *GPS*_*MFC*_ and clinical assessment scores, which would help determine whether adherence indicator-based assessments after each session can be utilized as a clinical assessment tool. Additionally, integrating this model with electronic health records could enhance clinical decision support. Prospective validation studies are essential to ensure its effectiveness in real-world settings. Furthermore, extending these approaches to other neurovascular conditions could lead to personalized treatments and continuous monitoring.

## References

[1] Gerstl JVE, Blitz SE, Qu QR, Yearley AG, Lassarén P, Lindberg R, et al. Global, regional, and national economic consequences of Stroke. Stroke. 2023;54(9):2380–9. doi: 10.1161/STROKEAHA.123.043131 37497672 PMC7614992

[2] Roelofs JMB, Zandvliet SB, Schut IM, Huisinga ACM, Schouten AC, Hendricks HT, et al. Mild stroke, serious problems: limitations in balance and gait capacity and the impact on fall rate, and physical activity. Neurorehabil Neural Repair. 2023;37(11–12):786–98. doi: 10.1177/15459683231207360 37877724 PMC10685695

[3] Nagano H, Prokofieva M, Asogwa CO, Sarashina E, Begg R. A machine learning model for predicting critical Minimum Foot Clearance (MFC) heights. Applied Sciences. 2024;14(15):6705. doi: 10.3390/app14156705

[4] Batchelor FA, Hill KD, Mackintosh SF, Said CM, Whitehead CH. Effects of a multifactorial falls prevention program for people with stroke returning home after rehabilitation: a randomized controlled trial. Arch Phys Med Rehabil. 2012;93(9):1648–55. doi: 10.1016/j.apmr.2012.03.031 22503739

[5] Dean CM, Rissel C, Sherrington C, Sharkey M, Cumming RG, Lord SR, et al. Exercise to enhance mobility and prevent falls after stroke: the community stroke club randomized trial. Neurorehabil Neural Repair. 2012;26(9):1046–57. doi: 10.1177/1545968312441711 22544817

[6] Nagano H, Said CM, James L, Sparrow WA, Begg R. Biomechanical correlates of falls risk in gait impaired stroke survivors. Front Physiol. 2022;13:833417. doi: 10.3389/fphys.2022.833417 35330930 PMC8940193

[7] Begg R, Galea MP, James L, Sparrow WAT, Levinger P, Khan F, et al. Real-time foot clearance biofeedback to assist gait rehabilitation following stroke: a randomized controlled trial protocol. Trials. 2019;20(1):317. doi: 10.1186/s13063-019-3404-6 31151480 PMC6545011

[8] Begg RK, Tirosh O, Said CM, Sparrow WA, Steinberg N, Levinger P, et al. Gait training with real-time augmented toe-ground clearance information decreases tripping risk in older adults and a person with chronic stroke. Front Hum Neurosci. 2014;8:243. doi: 10.3389/fnhum.2014.00243 24847234 PMC4021142

[9] Best R, Begg R. A method for calculating the probability of tripping while walking. J Biomech. 2008;41(5):1147–51. doi: 10.1016/j.jbiomech.2007.11.023 18255076

[10] Said CM, Galea M, Lythgo N. Obstacle crossing following stroke improves over one month when the unaffected limb leads, but not when the affected limb leads. Gait Posture. 2014;39(1):213–7. doi: 10.1016/j.gaitpost.2013.07.008 23916414

[11] Pathak P, Moon J, Roh S-G, Roh C, Shim Y, Ahn J. Application of vibration to the soles reduces minimum toe clearance variability during walking. PLoS One. 2022;17(1):e0261732. doi: 10.1371/journal.pone.0261732 34982783 PMC8726470

[12] Winter DA. Biomechanics and motor control of human gait: normal, elderly and pathological. 1991.

[13] Asogwa CO, Nagano H, Wang K, Begg R. Using deep learning to predict minimum foot-ground clearance event from toe-off kinematics. Sensors (Basel). 2022;22(18):6960. doi: 10.3390/s22186960 36146308 PMC9502804

[14] Nagano H. Gait Biomechanics for fall prevention among older adults. Applied Sciences. 2022;12(13):6660. doi: 10.3390/app12136660

[15] Nagano H, Said CM, James L, Begg RK. Feasibility of using foot-ground clearance biofeedback training in treadmill walking for post-Stroke gait rehabilitation. Brain Sci. 2020;10(12):978. doi: 10.3390/brainsci10120978 33322082 PMC7764443

[16] Khallaf ME, Gabr AM, Fayed EE. Effect of task specific exercises, gait training, and visual biofeedback on equinovarus gait among individuals with stroke: randomized controlled study. Neurol Res Int. 2014;2014:693048. doi: 10.1155/2014/693048 25538853 PMC4265373

[17] Teran-Yengle P, Cole KJ, Yack HJ. Short and long-term effects of gait retraining using real-time biofeedback to reduce knee hyperextension pattern in young women. Gait Posture. 2016;50:185–9. doi: 10.1016/j.gaitpost.2016.08.019 27637090

[18] Franz JR, Maletis M, Kram R. Real-time feedback enhances forward propulsion during walking in old adults. Clin Biomech (Bristol). 2014;29(1):68–74. doi: 10.1016/j.clinbiomech.2013.10.018 24238977

[19] Schenck C, Kesar TM. Effects of unilateral real-time biofeedback on propulsive forces during gait. J Neuroeng Rehabil. 2017;14(1):52. doi: 10.1186/s12984-017-0252-z 28583196 PMC5460355

[20] Santucci V, Alam Z, Liu J, Spencer J, Faust A, Cobb A, et al. Immediate improvements in post-stroke gait biomechanics are induced with both real-time limb position and propulsive force biofeedback. J Neuroeng Rehabil. 2023;20(1):37. doi: 10.1186/s12984-023-01154-3 37004111 PMC10064559

[21] Genthe K, Schenck C, Eicholtz S, Zajac-Cox L, Wolf S, Kesar TM. Effects of real-time gait biofeedback on paretic propulsion and gait biomechanics in individuals post-stroke. Top Stroke Rehabil. 2018;25(3):186–93. doi: 10.1080/10749357.2018.1436384 29457532 PMC5901660

[22] Thompson VU, Panchev C, Oakes M. Performance evaluation of similarity measures on similar and dissimilar text retrieval. In: 2015 7th International Joint Conference on Knowledge Discovery, Knowledge Engineering and Knowledge Management (IC3K). 2015. p. 577–84.

[23] Khandoker AH, Sparrow WA, Begg RK. Tone entropy analysis of augmented information effects on toe-ground clearance when walking. IEEE Trans Neural Syst Rehabil Eng. 2016;24(11):1218–24. doi: 10.1109/TNSRE.2016.2538294 27071178

[24] Khandoker AH, Lai DTH, Begg RK, Palaniswami M. Wavelet-based feature extraction for support vector machines for screening balance impairments in the elderly. IEEE Trans Neural Syst Rehabil Eng. 2007;15(4):587–97. doi: 10.1109/TNSRE.2007.906961 18198717

[25] Begg R, Best R, Dell’Oro L, Taylor S. Minimum foot clearance during walking: strategies for the minimisation of trip-related falls. Gait Posture. 2007;25(2):191–8. doi: 10.1016/j.gaitpost.2006.03.008 16678418

[26] Zhelezniak V, Savkov A, Shen A, Hammerla NY. Correlation coefficients and semantic textual similarity. arXiv preprint 2019. doi: arXiv:190507790

[27] Paliwal KK, Agarwal A, Sinha SS. A modification over Sakoe and Chiba’s dynamic time warping algorithm for isolated word recognition. Signal Processing. 1982;4(4):329–33. doi: 10.1016/0165-1684(82)90009-3

[28] Sakoe H, Chiba S. Dynamic programming algorithm optimization for spoken word recognition. IEEE Trans Acoust, Speech, Signal Process. 1978;26(1):43–9. doi: 10.1109/tassp.1978.1163055

[29] Warren Liao T. Clustering of time series data—a survey. Pattern Recognition. 2005;38(11):1857–74. doi: 10.1016/j.patcog.2005.01.025

[30] Pree H, Herwig B, Gruber T, Sick B, David K, Lukowicz P. On general purpose time series similarity measures and their use as kernel functions in support vector machines. Information Sciences. 2014;281:478–95. doi: 10.1016/j.ins.2014.05.025

[31] Cassisi C, Montalto P, Aliotta M, Cannata A, Pulvirenti A. Similarity measures and dimensionality reduction techniques for time series data mining. Advances in data mining knowledge discovery and applications. 2012. p. 71–96.

[32] Sengupta N, Begg R, Rao AS, Bajelan S, Said CM, Palaniswami M. Predicting improvement in biofeedback gait training using short-term spectral features from minimum foot clearance data. Front Bioeng Biotechnol. 2024;12:1417497. doi: 10.3389/fbioe.2024.1417497 39262630 PMC11387987

[33] Burges CJC. A tutorial on support vector machines for pattern recognition. Data Mining and Knowledge Discovery. 1998;2(2):121–67. doi: 10.1023/a:1009715923555

[34] Mitchell T. Decision tree learning. Machine learning. 1997;414:52–78.

[35] Breiman L. Random Forests. Machine Learning. 2001;45(1):5–32. doi: 10.1023/a:1010933404324

[36] Freund Y, Schapire RE. A decision-theoretic generalization of on-line learning and an application to boosting. Journal of Computer and System Sciences. 1997;55(1):119–39. doi: 10.1006/jcss.1997.1504

[37] Dietterich TG. An experimental comparison of three methods for constructing ensembles of decision trees: bagging, boosting, and randomization. Machine Learning. 2000;40(2):139–57. doi: 10.1023/a:1007607513941

[38] Haykin S. Neural networks and learning machines. 3 ed. Pearson Education India; 2009.

[39] Saripuddin M, Suliman A, Syarmila Sameon S, Jorgensen BN. Random undersampling on imbalance time series data for anomaly detection. In: 2021 The 4th International Conference on Machine Learning and Machine Intelligence. 2021. p. 151–6. 10.1145/3490725.3490748

[40] Hasanin T, Khoshgoftaar T. The effects of random undersampling with simulated class imbalance for Big Data. In: 2018 IEEE International Conference on Information Reuse and Integration (IRI). 2018. p. 70–9. 10.1109/iri.2018.00018

[41] Fernandez A, Garcia S, Herrera F, Chawla NV. SMOTE for learning from imbalanced data: progress and challenges, marking the 15-year anniversary. jair. 2018;61:863–905. doi: 10.1613/jair.1.11192

[42] Larsen BS. Synthetic Minority Over-sampling Technique (SMOTE); 2025. https://github.com/your_repo_link

[43] Chawla NV, Bowyer KW, Hall LO, Kegelmeyer WP. SMOTE: synthetic minority over-sampling technique. jair. 2002;16:321–57. doi: 10.1613/jair.953

[44] Murphy KP. Machine learning: a probabilistic perspective. MIT Press; 2012.

[45] Ding C, Peng H. Minimum redundancy feature selection from microarray gene expression data. J Bioinform Comput Biol. 2005;3(2):185–205. doi: 10.1142/s0219720005001004 15852500

[46] Christian J, Kröll J, Schwameder H. Comparison of the Classifier oriented gait score and the gait profile score based on imitated gait impairments. Gait Posture. 2017;55:49–54. doi: 10.1016/j.gaitpost.2017.04.007 28411445

[47] Ben Chaabane N, Conze P-H, Lempereur M, Quellec G, Rémy-Néris O, Brochard S, et al. Quantitative gait analysis and prediction using artificial intelligence for patients with gait disorders. Sci Rep. 2023;13(1):23099. doi: 10.1038/s41598-023-49883-8 38155189 PMC10754876

[48] Van Rijsbergen CJ. Information retrieval. 1979.

[49] Ghanem M, Ghaith AK, El-Hajj VG, Bhandarkar A, de Giorgio A, Elmi-Terander A, et al. Limitations in evaluating machine learning models for imbalanced binary outcome classification in spine surgery: a systematic review. Brain Sci. 2023;13(12):1723. doi: 10.3390/brainsci13121723 38137171 PMC10741524

[50] Akoglu H. User’s guide to correlation coefficients. Turk J Emerg Med. 2018;18(3):91–3. doi: 10.1016/j.tjem.2018.08.001 30191186 PMC6107969

